# Uptake and acceptability of human papillomavirus self-sampling in rural and remote aboriginal communities: evaluation of a nurse-led community engagement model

**DOI:** 10.1186/s12913-020-05214-5

**Published:** 2020-05-11

**Authors:** Tegan Dutton, Jo Marjoram, Shellie Burgess, Laurinne Montgomery, Anne Vail, Nichole Callan, Sunil Jacob, David Hawkes, Marion Saville, Jannine Bailey

**Affiliations:** 1 Western Sydney University, School of Medicine, Bathurst Rural Clinical School, PO Box 9008, Bathurst, NSW 2795 Australia; 2 Marathon Health, Bathurst, Australia; 3 Dubbo Medical and Allied Health Group, Dubbo, Australia; 4 Victorian Cytology Service Pathology, Melbourne, Australia

**Keywords:** Aboriginal women, Cervical screening, Program evaluation, Aboriginal Women’s business, Cancer

## Abstract

**Background:**

Aboriginal women experience disproportionately higher rates of cervical cancer mortality yet are less likely to participate in screening for early detection. This study sought to determine whether a community-based HPV self-sampling service model can effectively recruit never-screened and under-screened Aboriginal women to participate in cervical cancer screening; assess the clinical outcomes; and explore the acceptability of the model from the perspective of the participants.

**Methods:**

Aboriginal women aged 25–69 years of age were recruited from eight rural and remote communities in New South Wales, Australia to participate in HPV self-sampling via a community-based service model. Outcome measures were: number of women screened by HPV self-sampling, their prior cervical screening status (under-screened or never-screened), clinical outcomes and participation in follow-up pathways of care, and satisfaction with the service model.

**Results:**

In total, 215 women conducted a HPV self-sampling test and 200 evaluation surveys were completed. One-fifth of participants (*n* = 46) were never-screened and one-third (*n* = 69) were under-screened. Many were unsure of their screening status. Nine women were HPV 16/18 positive and eight had completed all follow up by the conclusion of the study. A further 30 women tested positive for a high risk type other than HPV 16/18 (HPV other), of which 14 had completed follow up at the conclusion of the study. Satisfaction with the HPV self-sampling kit, the process of self-sampling and the service model was high (> 92% satisfied on all items). Many women had difficulty understanding their official HPV results and placed high importance on the nurse explaining it to them.

**Conclusions:**

A community-based service model that respects Aboriginal Women’s Business can effectively recruit under-screened and never-screened Aboriginal women to complete cervical cancer screening. Furthermore, this service model supports them to complete recommended follow-up care and engage with their local existing health services.

## Background

In December 2017, the Australian National Cervical Screening Program (NCSP) was updated, with HPV testing replacing Pap tests. Other changes include an increase in entry age from 18 to 25 years, and recommended screening every five years (previously two years). Also, clinician-supervised, HPV self-sampling became available to eligible women. To be eligible for self-sampling, a woman must be aged 30 years or over and be overdue for screening by at least two years [1]. This change was based on extensive evidence demonstrating that HPV testing is a more sensitive screening test, providing better protection against cervical cancer through detection of HPV before cell changes occur in the cervix [2–4]. Key to the NCSP’s success however, is in effectively recruiting women to participate in screening. Never-screened or under-screened women are at higher risk of cervical cancer than those who regularly participate in screening programs [5–7].This recent change to HPV testing and the ability to self-sample provides a unique opportunity to engage these women.

National cervical screening rates for Aboriginal and Torres Strait Islander women is unknown as pathology forms do not record Indigenous status. However, several studies that have utilised community level and State/Territory data have indicated that the average screening rates for Indigenous women lies between 33.5 and 44%, compared to 55.7–59.1% for non-Indigenous women… [8–12] Aboriginal and Torres Strait Islander women also experience higher rates of cervical cancer incidence and mortality [12, 13], hence, increasing their participation in preventative screening is crucial.

International research aimed at increasing the uptake of cervical screening was summarised in a recent meta-analysis [14]. The study found that door-to-door recruitment was most effective at increasing screening uptake as compared to clinician-collected samples. This was followed by community campaigns and mail-out to all, with the opt-in approach considered ineffective. However, the success of these recruitment techniques varied across populations and geographical regions, and thus techniques need to be tailored to specific regions. The best way to offer self-sampling to women who are disengaged from the health system and are under- or never-screened is also not yet known. To our knowledge, no other programs in Australia have offered face-to-face distribution and collection of HPV self-sampling kits within the home and community. One Australian study successfully recruited Aboriginal women from a regional Aboriginal Community Controlled Health Service to participate in self-sampling [15], suggesting self-sampling is an acceptable option for Aboriginal and Torres Strait Islander women. No HPV self-collection studies have specifically targeted Aboriginal and Torres Strait Islander women in rural and remote communities.

The option of clinician-supervised patient self-sampling under the new NCSP could positively impact screening practices of women who have never been screened or are under-screened. However, this pathway relies on existing patient engagement with a clinician and/or health service. If a woman does not routinely engage with a primary healthcare service, opportunities to actively encourage screening via this pathway are limited. This is particularly important in rural and remote communities where access to female health professionals can be limited and the proportion of under-screened and never-screened women increases. Innovative models of care are needed to engage these hard-to-reach women. This research sought to: determine whether a community based HPV self-sampling model effectively recruited never-screened and under-screened Aboriginal women to participate in cervical cancer screening; assess the clinical outcomes, including follow-up; and explore the acceptability of the model from the perspective of the participants.

## Methods

### Community consultation

Marathon Health Primary Health Care Nurses (PHCNs) consulted extensively with local organisations and community members across rural and remote New South Wales, Australia to establish the new service model for HPV self-sampling amongst Aboriginal women, including clinical governance processes.

### Pilot sites

**Fig. 1:**
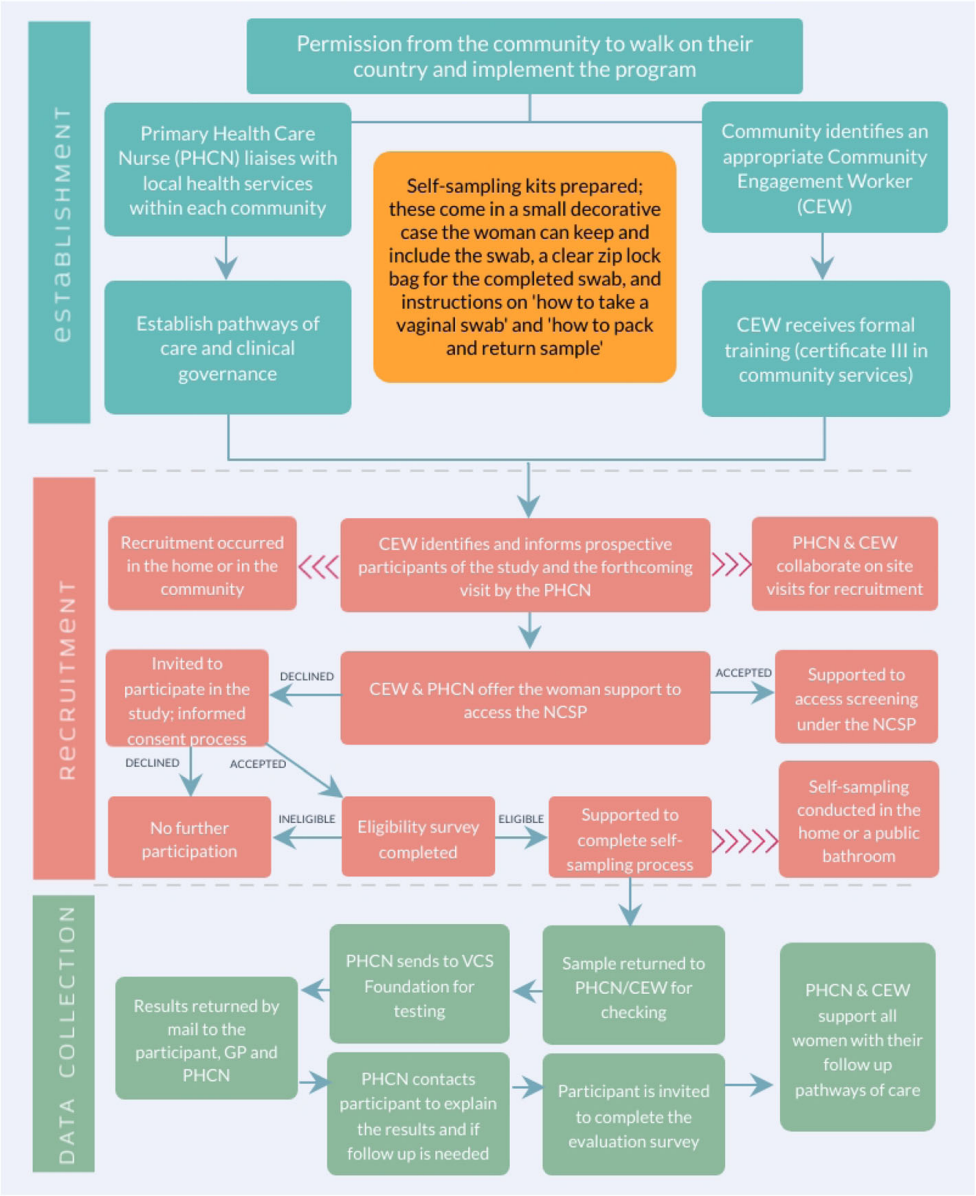
Study flowchart

Eight rural and remote communities participated in the pilot study. Notably, seven of these had minimal access to female General Practitioners (GPs). Our target sample size (*n* = 266) was calculated based on the estimated number of Aboriginal women who were under-screened. Across the study region in 2011, the total female Aboriginal population was 596 and an estimated 45% of women (both Aboriginal and non-Aboriginal) were under-screened (data provided by the NSW Cancer Institute, 2011–13). *Self-sampling pathway:* PHCNs established partnerships with Local Aboriginal Land Councils (LALC) as the representative body for Aboriginal people in each community. Each LALC identified a female employee or community member to recruit as the Community Engagement Worker (CEW) for each site. Their primary role was to engage and recruit women to the program, and support them along the self-sampling pathway, with clinical support provided by the PHCN. Once the women completed the self-sample with the support of the PHCN and CEW, the PHCN sent the HPV samples to the VCS Foundation to be analysed. Pathology results were returned by mail to the participant, their nominated GP and the PHCN. A project GP was recruited to provide clinical oversight of those participants who did not have a regular GP. The PHCN followed up with all participants face-to-face or by phone to ensure they received their results and that they understood their meaning, including any follow-up that was required. Women who returned invalid results were provided the option to rescreen. See Fig. 1 for a detailed flowchart of the self-sampling process.

### Recruitment of women

The CEWs were members of the local Aboriginal community themselves, and had existing links with the community to identify and engage with local women about the program. Whilst some women were recruited at local community events, the majority were recruited via home visits. Convenience sampling was used to recruit women, followed by snowballing (referred to as the “Koori Grapevine” by the local Aboriginal women) with the women identifying other family and friends for cervical screening. Whilst this inevitably would have resulted in bias, gaining trust with the community and having the women take on some ownership increased acceptance of the program. .

### Participants

The renewed NCSP recommends clinician-led HPV screening for women in the age range 25–69 years (inclusive), therefore our study included women in this age range. It is worth noting however, that the current NCSP only permits self-sampling to be completed at 30 years of age, when a woman is at least 5 years overdue for screening. Screening of women younger than 25 years was allowed at the discretion of the PHCN based on clinical indication (for example, sexual debut younger than 14 years of age and have not received the HPV vaccination). The NCSP recommends all women conduct an ‘exit-screen’ between 70 and 74 years of age. We elected not to include women in this age group so as to ensure they conducted their recommended exit screen outside of this pilot study under the guidance of their clinician.

Whilst we sought to recruit women who were under-screened, the CEW and PHCN were able to decide whether they believed it was clinically and ethically appropriate to screen women who had been screened in the previous 4 years, particularly if they were due for a screen or were overdue.

### Data collection and management

An eligibility survey was conducted at the outset of the recruitment process. An evaluation survey was conducted during follow-up to elicit feedback on the service model and the participant’s experience of self-sampling using likert scale responses from 1 (very unsatisfied) to 5 (very satisfied). Clinical data were input into the client’s electronic medical record as per routine practice. All data were stored securely and confidentially so as to comply with both clinical governance requirements and research ethics requirements.

All data were linked using a code number and then de-identified by the PHCN before analysis by the research team. Data were entered into Excel. IBM SPSS Statistics (version 22) was used for descriptive statistics on the quantitative dataset. Open-ended comments were combined and key themes then extracted by two researchers independently as part of the manifest content analysis process [16]. Themes and sub-themes were compared and discussed until a consensus was reached.

### Ethics approval

Ethics approval was granted by the Aboriginal Health and Medical Research Council Human Research Ethics Committee (reference 1188/16) and Western Sydney University Human Research Ethics Committee (reference H11837).

## Results

### Part 1: clinical results

**Fig. 2:**
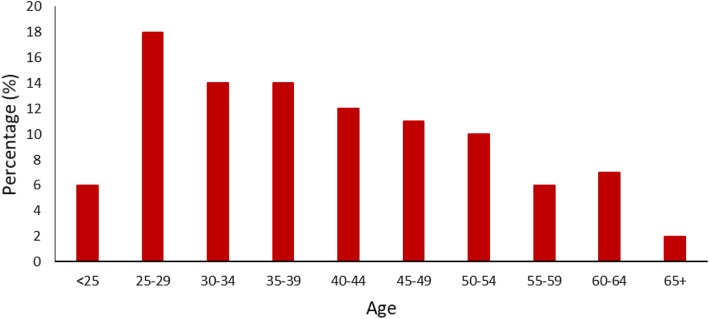
Age distribution of women that participated in HPV self-sampling

HPV self-sampling kits were distributed from September 2016 to June 2018 (21 months). The 215 women (80.8% of the targeted sample) that agreed to participate were all eligible and completed the HPV self-sample. Participation was highest amongst women aged 25–29 years (Fig. 2).

One-fifth of participants (21.4%, 46) self-reported that they had never completed a Pap test and 32.1% (69) had previously completed a Pap test but more than 4 years ago, with the range since last Pap test being 4 to 20 years. A total of 26.0% (56) of participating women reported that they had been screened, but could not remember when, suggesting that it was more than 4 years ago. Of the 34 (15.8%) women that had been screened in the previous 4 years, all but thirteen (6%) had not been screened in the previous two years, and thus were due for screening. Last screen was not recorded or was unknown for 10 (4.7%) participants.

Eighteen percent (18.1%; 39) of women had a positive result: 4.2% [9] were HPV Positive 16/18 and 14% (30) HPV Positive other (defined as a high risk HPV type other than 16 or 18). Seventy-eight percent (78%, 168) had a negative result (two of which were the results of a second screen as the initial result was invalid), and 3.7% [8] had invalid results *and* did not complete a rescreen. Stratification by age group showed that women who tested positive were distributed across all of the age groups (Table 1).

**Table 1:** HPV test results stratified by age group

HPV Test Result	Negative	Positive for HPV 16/18 type	Positive for a type other than HPV 16/18
*Age Group*			
< 25 years	8 (4.8%)	0	5 (12.8%)
25–29 years	27 (16.1%)	1 (11.1%)	10 (25.6%)
30–34 years	27 (16.1%)	1 (11.1%)	4 (10.3%)
35–39 years	22 (13.1%)	2 (22.2%)	6 (15.4%)
40–44 years	20 (11.9%)	0	5 (12.8%)
45–49 years	19 (11.3%)	2 (22.2%)	3 (7.7%)
50–54 years	18 (10.7%)	1 (11.1%)	2 (5.1%)
55–59 years	11 (6.5%)	0	2 (5.1%)
60–64 years	12 (7.1%)	2 (22.2%)	2 (5.1%)
65 years and over	4 (2.4%)	0	0

**Fig. 3:**
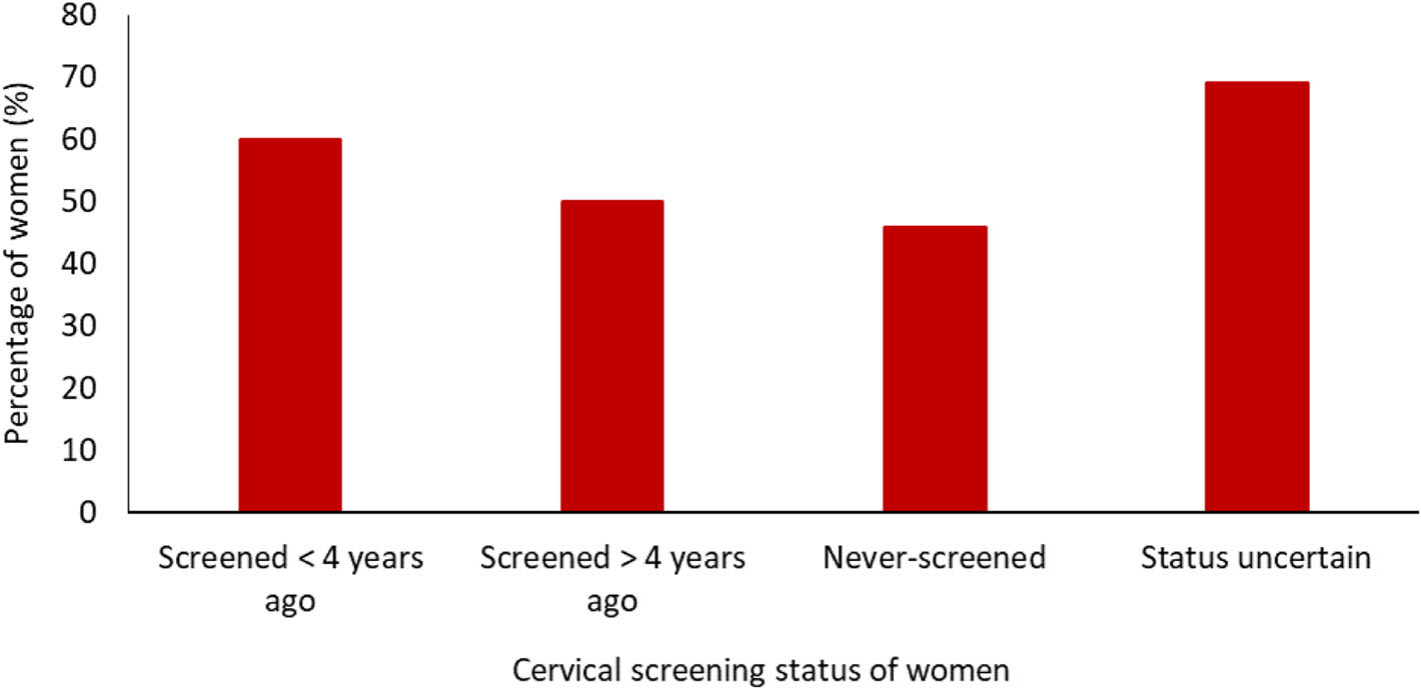
Follow-up rate of women who tested positive for HPV by cervical screening status

Eight of the nine women (88.9%) with a HPV Positive 16/18 result had attended a colposcopy appointment by mid-July 2018. The other woman failed to attend her appointments due to documented clinical and other issues. Just under half (46.7%; 14) of the women with a HPV Positive other result had attended an appointment for a cervical screen by mid-July 2018 when this study ended. Attendance at follow-up appointments was relatively similar regardless of cervical screening history (Fig. 3).

### Part 2: Women’s satisfaction and feedback

Almost all women (92.6%; 199) completed the follow-up evaluation survey. One completed the survey twice as her initial result was invalid (200 total surveys). More than 90% of women were highly satisfied with the HPV self-sampling kit and the process involved (Table 2). In response to whether results were presented in an easy to understand format, 13.2% (23) were unsatisfied or very unsatisfied. Further, some women that were highly satisfied with the format of results commented that they were unable to interpret the paper copy independently, but the PHCN explained the results well:

**Table 2:** Participating women’s level of satisfaction with the HPV self-sampling program

Aspects of the self-sampling program	(5) very satisfied	(4) satisfied	(3) neither satisfied/ dissatisfied	(2) dissatisfied	(1) very dissatisfied
*1.1 HPV self-sampling kit*					
The kit provided everything to complete the self-sampling test? (*n* = 200)	199 (99.5%)	1 (0.5%)	–	–	–
Satisfaction with the self-sampling instructions (n = 200)	196 (98.0%)	1 (0.5%)	1 (0.5%)	–	2 (1%)
*1.2 HPV self-sampling process*					
Process clearly explained by the CEW (*n* = 197)	188 (95.4%)	5 (2.5%)	3 (1.5%)	1 (0.5%)	–
Able to ask questions and receive answers in a timely manner? (*n* = 186^a^)	183 (98.4%)	2 (1.1%)	–	1 (0.5%)	–
The process was simple (n = 197)	187 (94.9%)	4 (2%)	4 (2%)	–	2 (1.0%)
Provided with privacy and confidentiality (n = 197)	182 (92.4%)	6 (3.0%)	7 (3.6%)	2 (1.0%)	–
*1.3 Results*					
Results were provided in an easy to understand format (*n* = 174^b^)	136 (78.2%)	6 (3.4%)	9 (5.2%)	3 (1.7%)	20 (11.5%)
	< 2 weeks	< 3 weeks	> 3 weeks	Unsure	Not at time PHCN made contact^c^
Mailed results returned within 2 weeks (*n* = 195)	148 (75.9%)	13 (6.7%)	6 (3.1%)	13 (6.7%)	15 (7.7%)

^a^ Eight participants said they did not need to ask questions and six did not respond to this question

^b^ Some participants had not yet received results and thus not applicable or did not respond to this question

^c^ The hard copy paper results were not yet received by the participant at the time the PHCN provided the results verbally over the phone (this may have been within two weeks of the self-sampling test)

*“The pathology made no sense to me but when the Nurse called and discussed I understood them”.*

The majority of participants (96.0%; 192) would use the HPV self-sampling kit again. Of the six women who stated they would not use it again, four were referred to the GP for a pap test and would thus go directly to the GP next time, and two experienced difficulties completing the sample. Almost all women (98.5%; 197) would recommend the HPV self-sampling kit to other women.

Women were grateful of, and highly satisfied with, the service (Table 3). There was overwhelming agreement that self-sampling removed the shame, intimidation, embarrassment, and pain that has historically been associated with clinician-collected Pap tests. Positive aspects included the ability to complete the test in the home, and thus the accessibility and privacy of cervical screening; the simplicity of the test; being in charge of Women’s business (an important consideration in Aboriginal culture where certain aspects of life are performed separately and termed Men’s and Women’s business [17] – cervical screening falls into this category); the appropriateness of the self-sampling kit contents, with the exception of there not being a non-transparent bag to return the swab in; and the professionalism of the PHCN. Women felt they would not have completed cervical screening had this service not been available.

**Table 3:** Participating women’s experiences with HPV self-sampling

Themes	Description
Location of self-sample	In the home: Women felt it was a more private, confidential and comfortable experience; they could complete it without embarrassment and shame.
	Out of the home: There was mixed feelings regarding privacy and confidentiality when completed out of the home (e.g. park, LALC, mother’s groups).
Accessible	Accessible (in the home) and free. No need to travel long distances for a female GP, or wait and pay at a General Practice.
Privacy and confidentiality – shame and embarrassment	Self-sampling was private and confidential, with no shame or embarrassment. Women did not feel violated or lose their dignity.
Simplicity	Self-sampling was described as simple, easy, convenient, quick, and not too daunting. Several women experienced some difficulty completing the test themselves.
In charge of Aboriginal women’s business^a^	Women felt a sense of control over their own women’s business, health and wellbeing. Women found it to be a positive and personal experience, and they felt comfortable and at ease.
Comparison of HPV self-sampling to Pap test	*Pap test* – uncomfortable, painful, humiliating, daunting, shameful, embarrassing, degrading, intimidating, not confidential. Women do not like going to the Doctor and do not want a man involved in women’s business. *HPV self-sampling* – easier, more private, discreet, comfortable, personal, dignified, appropriate, and quicker. Not as intrusive, evasive or awkward.
Self-sampling kit contents	High importance was place on the quality of the instruction cards: (a) They were clear, easy to understand, straight forward – not having to sift through unnecessary readings. (b) Illustrations for black women were good. In the case a woman could not read, the Nurse could clearly explain the process and the picture cards supported this.
	The women were happy they could keep the case the kit came in.
	A non-transparent bag needs to be included in the kit for the sample to be returned to improve privacy and remove any shame.
The PHCN gave the women confidence in the service being professional	Women placed high importance on a trained professional (i.e. the PHCN) being present throughout the program, giving them confidence that it was accurate and professional. Some women would have preferred the PHCN (instead of the CEW) to explain the kit/process and take the completed sample.
Verbal communication of results	Women appreciated the verbal communication of results from the PHCN, particularly as many women could not understand them. This was described as ‘caring’ and gave women confidence in the entire program.
Unlikely that women would have completed a cervical screen had the HPV self-sampling test not been offered	Women commented on: never having completed a cervical screen until participating in this study; being hesitant, reluctant, frightened, or scared to go to the Doctor to have a cervical screen, even in the case that one woman had a family history of cervical cancer and understood the risks; postponing Pap tests, with women suggesting anywhere between 1 and 20 years before they thought they would be screened again.
Grateful of a potential lifesaving experience	Women (irrelevant of results) were grateful and happy they had been offered the self-sampling kit, and that it could have saved their lives.
Initial concerns with the Program	Women were not always forthcoming to completing the self-sampling, describing that they were initially nervous (e.g. unsure of accuracy of the test; previous painful experience with a pap test; worried of a positive result). However, these comments were followed by positive feedback about the test and increased confidence to complete the test next time.

^a^ In Aboriginal culture, certain aspects of life are performed separately for men and women, and are termed Men’s business and Women’s business [17]; cervical screening and related items fall into this category

*“I could do it myself. No pain. I ended up having to go to the GP for a Pap test but I would have never done that if I didn’t have a self-sample, may have saved my life. I recommend this to everyone. The government needs to give all women the choice to self-sample”.*

## Discussion

This program successfully recruited never- and under-screened Aboriginal women across rural and remote Australia to participate in HPV self-sampling in the home or community. Indeed, we were able to recruit 81% of our target sample size to participate in screening, a value much larger than the estimated 30–40% of Aboriginal women who have traditionally been recruited to participate in cervical screening [8–12]. Certainly, one-fifth of participating women had never been screened previously, one-third were overdue for screening, and a further quarter could not remember when they were last screened, suggesting it was a long time ago and they were therefore likely to be overdue for screening.

Despite historically being disengaged from the health system, eight of the nine women with a HPV Positive 16/18 had attended a colposcopy appointment by the completion of the project. This is in line with other studies that also reported high follow-up rates for women who participated in self-sampling [13, 15]. The lower follow-up rates (46.7%) observed for women who tested HPV Positive Other (defined as a high risk HPV type other than HPV 16 or 18) may be the result of not tracking women for long enough as anecdotal evidence provided after the completion of this study reported that some women did attend a follow up GP appointment that occurred after the end date of this study. Long-term research is therefore needed to understand whether follow-up care is achieved and how women can best be supported.

Given the target population was not actively engaged in cervical cancer screening or the health system, the timely and individual support the PHCN and CEW provided to each woman to ensure results and follow-up care were understood, and women remained engaged, was key to the model’s success. Women placed high importance on the verbal communication of results given by the PHCN, particularly as they did not understand the hard copy results. Ensuring women have maximum understanding of the next steps in their pathway of care decreases anxiety and assists with successful completion of the self-sampling pathway [18]. Flexibility in PHCN and CEW roles enabled innovative solutions to be adapted for each woman to support them at an individual level to access the follow-up care required. This included offering self-sampling in the home environment. The majority of self-sampling studies have not allowed for home-based self-collection so this study adds to the limited evidence base attesting to its effectiveness at engaging women to complete screening.

There was overwhelming agreement that this method for cervical cancer screening is appropriate for Aboriginal women, ensuring Aboriginal Women’s Business is respected. In comparison to Pap tests it was seen as a positive experience which women were happy to repeat and recommend to others, also documented in numerous self-sampling trials [18–21]. Importantly, women mentioned they would not have participated in screening had this not been available.

This pilot highlighted how the model can provide an access point into the health system, demonstrated by the large proportion of women that entered the mainstream health system for follow-up care. Further, an unexpected outcome was that some women who attended a GP follow-up appointment, also sought after additional medical advice/treatment for other conditions. It provided a comfortable access point for these women, and therefore can have a net overall positive impact on health service engagement.

Whilst the current NCSP supports never- and under-screened women to participate in self-sampling rather than clinician-led sampling, there are specific criteria and it must be facilitated and requested by a clinician who routinely performs cervical screening. Hence, it is reliant on existing engagement with a clinician/health service. The current community-based service model could serve as an effective adjunct to clinician-led NCSP services by engaging with those women who are currently not engaged with a health service or clinician. Significantly, data from this study shows that this service model can also facilitate health service engagement amongst women who were previously disengaged. For many women though, the barriers to engagement with cervical screening under the previous NCSP will not immediately be broken down by completing one self-sample HPV test. It is a key recommendation of this study that women who are eligible for self-sampling continue to remain eligible to screen via this pathway, should they decline clinician-led screening in the future.

## Limitations

This study did not capture numbers of women who were approached and declined participation or were obviously ineligible, such as being pregnant. Screening history was self-reported and thus there may have been some error in this data. There was not a structured process in place to document the follow-up outcomes for women and support required, including a formal clinical record review to determine actual disease burden from biopsy results. When women completed the evaluation survey there was a feeling that using a scale of 1 to 5 was not always understood by the women, and that a yes/no response (or something similar) would be more appropriate. The data collection timeframe was longer than anticipated (21 months) and this was due to a number of external factors along with workforce challenges in retaining CEWs in some of the communities. For reasons unrelated to the project, a number of CEWs relocated to other regions and new CEWS had to be recruited. Nonetheless, these are important considerations when scaling up services such as this across the region, particularly in rural and remote areas where health workforce is already a challenge.

## Conclusions

This pilot demonstrated how a community-based model that respects Aboriginal Women’s Business, is community-led, and has a clinical lead to maintain high clinical governance and professionalism within the program, can effectively recruit hard-to-reach under-screened and never-screened Aboriginal women to complete cervical cancer screening. This was evidenced through high screening participation rates, high levels of satisfaction amongst participating women and the high rates of women engaging with recommended follow-up care. It is extremely unlikely that these women would have engaged in the current NCSP via clinician-supervised self-sampling as they are not engaged in the health system (i.e. they do not visit GPs). Future research and evaluation is needed to explore translation of this best practice service model into other remote, rural and regional sites. Research that builds on the current pilot study data can inform the requirements of rolling out this service model to communities that differ by population size, remoteness, geography, health service access, and other aspects unique to that community. Long-term follow-up of HPV self-sampling participants can also shed light on the ongoing health service engagement and cervical screening practices of these women.

## Data Availability

The datasets are available from the corresponding author upon request.

## Abbreviations

CEW: Community Engagement Worker
GP: General Practitioner
HPV: Human Papilloma Virus
LALC: Local Aboriginal Lands Council
NCSP: National Cervical Screening Program
PHCN: Primary Health Care Nurse

## References

[1] Australian Government Department of Health. National Cervical Screening Program. Updated 28 Aug 2019. http://www.cancerscreening.gov.au/internet/screening/publishing.nsf/Content/cervical-screening-1 (viewed Aug 2019).

[2] Elfstrom KM, Smelov V, Johansson AL (2014). Long term duration of protective effect for HPV negative women: follow-up of primary HPV screening randomised controlled trial. BMJ..

[3] Ronco G, Dillner J, Elfstrom KM (2014). Efficacy of HPV-based screening for prevention of invasive cervical cancer: follow-up of four European randomised controlled trials. Lancet..

[4] Canfell K, Saville M, Caruana M (2018). Protocol for compass: a randomised controlled trial of primary HPV testing versus cytology screening for cervical cancer in HPV-unvaccinated and vaccinated women aged 25–69 years living in Australia. BMJ Open.

[5] VCS Foundation. Statistical Report 2015. 2017. https://www.vcs.org.au/population-health/statistical-reports/annual-statistical-reports/ (viewed Aug 2019).

[6] Bos AB, Rebolj M, Habbema JD (2006). Nonattendance is still the main limitation for the effectiveness of screening for cervical cancer in the Netherlands. Int J Cancer.

[7] Sasieni PD, Cuzick J, Lynch-Farmery E (1996). The national co-ordinating network for cervical screening working group. Estimating the efficacy of screening by auditing smear histories of women with and without cervical cancer. Br J Cancer.

[8] Australian Institute of Health and Welfare (2018). Cervical Screening in Australia 2018.

[9] Australian Institute of Health and Welfare (2017). National Key Performance Indicators for Aboriginal and Torres Strait Islander primary health care: results from June 2016.

[10] Binns PL, Condon JR (2006). Participation in cervical screening by indigenous women in the Northern Territory: a longitudinal study. MJA..

[11] Coory MD, Fagan PS, Muller JM (2002). Participation in cervical cancer screening by women in rural and remote aboriginal and Torres Strait islander communities in Queensland. MJA..

[12] Whop LJ, Garvey G, Baade P (2016). The first comprehensive report on indigenous Australian women's inequalities in cervical screening: a retrospective registry cohort study in Queensland, Australia (2000-2011). Cancer..

[13] Australian Institute of Health and Welfare. Australian Cancer Incidence and Mortality (ACIM) books: cervical cancer. 2017. https://www.aihw.gov.au/reports/cancer/cancer-data-in-australia/acim-books (viewed Aug 2019).

[14] Arbyn M, Smith SB, Temin S (2018). Detecting cervical precancer and reaching underscreened women by using HPV testing on self samples: updated meta-analyses. BMJ..

[15] Saville M, Hawkes D, McLachlan E (2018). Self-collection for under-screened women in a National Cervical Screening Program: pilot study. Curr Oncol (Toronto, Ont).

[16] Bengtsson M (2016). How to plan and perform a qualitative study using content analysis. NursingPlus Open.

[17] Barclay L, Andre CA, Glover PA (1989). Women’s business: the challenge of childbirth. Midwifery..

[18] Gupta S, Palmer C, Bik EM (2018). Self-Sampling for Human Papillomavirus Testing: Increased Cervical Cancer Screening Participation and Incorporation in International Screening Programs. Front Public Health.

[19] Madzima TR, Vahabi M, Lofters A (2017). Emerging role of HPV self-sampling in cervical cancer screening for hard-to-reach women: focused literature review. Canadian Family Physician Medecin de famille canadien.

[20] Sultana F, Mullins R, English DR (2015). Women’s experience with home-based self-sampling for human papillomavirus testing. BMC Cancer.

[21] Winer RL, Gonzales AA, Noonan CJ (2016). Assessing acceptability of self-sampling kits, prevalence, and risk factors for human papillomavirus infection in American Indian women. J Community Health.

